# Structural studies of β-glucosidase from the thermophilic bacterium *Caldicellulosiruptor saccharolyticus*

**DOI:** 10.1107/S2059798324009252

**Published:** 2024-10-01

**Authors:** Anastasia I. Sotiropoulou, Dimitris G. Hatzinikolaou, Evangelia D. Chrysina

**Affiliations:** a Institute of Chemical Biology National Hellenic Research Foundation 48 Vassileos Constantinou Avenue 116 35Athens Greece; b Enzyme and Microbial Biotechnology Unit, Department of Biology National and Kapodistrian University of Athens Panepistimiopolis Zografou 157 72Athens Greece; University of Oxford, United Kingdom

**Keywords:** biocatalysis, β-glucosidases, *Caldicellulosiruptor saccharolyticus*, X-ray crystallography

## Abstract

β-Glucosidase from the thermophilic bacterium *Caldicellulosiruptor saccharo­lyticus* (Bgl1) has been denoted as having an attractive catalytic profile for various industrial applications. Bgl1 catalyses the final step of in the decomposition of cellulose, an unbranched glucose polymer that has attracted the attention of researchers in recent years as it is the most abundant renewable source of reduced carbon in the biosphere. With the aim of enhancing the thermostability of Bgl1 for a broad spectrum of biotechnological processes, it has been subjected to structural studies. Crystal structures of Bgl1 and its complex with glucose were determined at 1.47 and 1.95 Å resolution, respectively. Bgl1 is a member of glycosyl hydrolase family 1 (GH1 superfamily, EC 3.2.1.21) and the results showed that the 3D structure of Bgl1 follows the overall architecture of the GH1 family, with a classical (β/α)_8_ TIM-barrel fold. Comparisons of Bgl1 with sequence or structural homologues of β-glucosidase reveal quite similar structures but also unique structural features in Bgl1 with plausible functional roles.

## Abstract 2

Crystal structures of a β-glucosidase from the thermophilic bacterium *C. saccharolyticus* (Bgl1) and its complex with glucose were determined at 1.47 and 1.95 Å resolution, respectively. Comparison of Bgl1 with sequence or structural homologues showed that Bgl1 is quite similar except for two regions, one of which seems to be a unique insertion of residues that is only present in Bgl1.

## 1. Introduction

**Figure 1:**
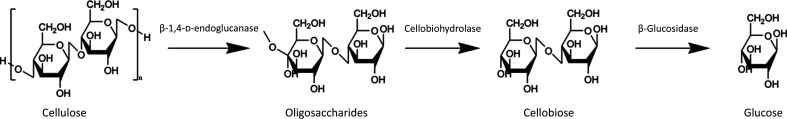
Schematic representation of the enzymatic hydrolysis of cellulose.

Cellulose is an abundant polysaccharide that is found in plant cell walls and is a key contributor to their rigidity. It is exploited as a source for environmentally friendly applications; thus, there is sustained interest in its optimal degradation, especially under the harsh conditions usually employed in industrial applications. To this end, there is a quest to optimize the cooperative action of the hydrolytic enzymes that target cellulose. Amorphous cellulose chains are randomly attacked by β-1,4-d-endoglucanase, producing oligosaccharides. Cellobiohydrolase, in turn, releases cellobiose from the reducing ends of cellulose, and finally β-glucosidase hydrolyses cellobiose to glucose, completing the overall bio­degradation process (Fig. 1 ▸).

β-Glucosidases (EC 3.2.1.21) are promising targets for exploitation as biocatalysts since they have been implicated in a wide range of industrial applications that go beyond their hydrolytic roles on cellobiose and β-1,4-oligosaccharides (Kannan *et al.*, 2023 ▸; Ouyang *et al.*, 2023 ▸); however, their poor thermostability remains a challenge. According to the Carbohydrate-Active enZYmes Database (CAZY; https://wwww.cazy.org/; Drula *et al.*, 2022 ▸), β-glucosidases span GH families 1–5 (GH1–5), with most being found in the GH1 family. In fact, more than 60 000 enzymes from archaea, bacteria and eukaryota belong to the GH1 family, of which only 349 have been characterized and 82 have had their three-dimensional structure determined by protein X-ray crystallo­graphy.

Microorganisms are the most widely used source of industrially relevant enzymes. The most common thermoresistant organisms identified from the β-glucosidase structures that have been deposited in the Protein Data Bank in Europe (PDBe; https://www.ebi.ac.uk/pdbe/; Armstrong *et al.*, 2019 ▸) and published in scientific journals include *Halothermothrix orenii* strain H 168 (UniProt Accession No. B8CYA8_HALOH), *Thermoanaerobacterium saccharolyticum* strain JW/SL-YS485 (I3VXG7_THESW), *Thermotoga maritima* strain MSB8 (BGLA_THEMA), *T. neapolitana* strain DSM 4359 (BGLA_THENN and Q0GC07_THENN), *Thermus nonproteolyticus* (Q9L794_9DEIN) and *T. thermophilus* strain HB8 (Q53W75_THET8). β-Glucosidases adopt the (β/α)_8_ TIM-barrel fold and their catalytic action is performed by the interaction of two conserved glutamic acid residues, one of which acts as a catalytic proton donor and the other as the catalytic nucleophile/base (Sharma *et al.*, 2019 ▸; Chen *et al.*, 2021 ▸). Very recently, a review by Mól and coworkers summarized the efforts made to date to immobilize β-glucosidase, the support materials used and their application, underlining the growing interest in this biologically important hydrolytic enzyme and the challenge in addressing its cost-effectiveness as an immobilized biocatalyst on an industrial scale (Mól *et al.*, 2023 ▸). Previous studies of thermophilic bacteria also revealed that *Caldicellulosiruptor saccharolyticus* could successfully be used for the production of β-glucosidase (Bgl1; UniProt Accession No. P10480; Hong *et al.*, 2009 ▸). Bgl1 belongs to the GH-A clan of the GH1 family (EC 3.2.1.21) based on its amino-acid sequence similarity and biochemical characteristics (Henrissat & Bairoch, 1993 ▸). Bgl1 has a half-life of 250 h at 60°C and it has been shown to be a thermostable enzyme with a broad substrate specificity and saccharification ability that efficiently hydrolyses cellooligosaccharides to glucose (Hong *et al.*, 2009 ▸). Here, we report the three-dimensional structures of Bgl1 from the thermophilic bacterium *C. saccharolyticus* and its complex with glucose, which is the product of its catalytic action when lactose is used as a substrate, that were determined at 1.47 and 1.95 Å resolution, respectively.

## 2. Materials and methods

### 2.1. Expression and purification of recombinant Bgl1

Genomic DNA from *C. saccharolyticus* DSM 8903 (Hong *et al.*, 2009 ▸) was used as the template for enzyme production. The Bgl1 open reading frame was cloned in pET-15b vector. His-tagged recombinant Bgl1 was expressed in *Escherichia coli* BL21(DE3) cells and was purified using immobilized metal-ion affinity chromatography (IMAC) as described previously (Galanopoulou *et al.*, 2016 ▸). The IMAC product was treated with thrombin to remove the His-tag and finally purified by gel filtration on a Sephacryl S-200 column to remove the thrombin and the His-tag part. The purity of the samples was assessed by SDS–PAGE. A single band corresponding to a molecular mass of 53.25 kDa was observed, indicating that the sample was sufficiently pure to be subjected to crystallization trials.

### 2.2. Enzymatic assay and thermostability

The hydrolytic activity and thermoresistance of Bgl1 were determined as described previously (Galanopoulou *et al.*, 2016 ▸). The enzymatic activity was determined first at 65°C and then at temperatures ranging from 40 to 75°C using *p*-nitrophenyl-β-d-glucopyranoside (pNP-G; purchased from Sigma–Aldrich) as a substrate at pH 6.5. The extent of hydrolysis was calculated by measuring the absorbance of pNP-G at 410 nm.

### 2.3. Sequence analysis

**Figure 2:**
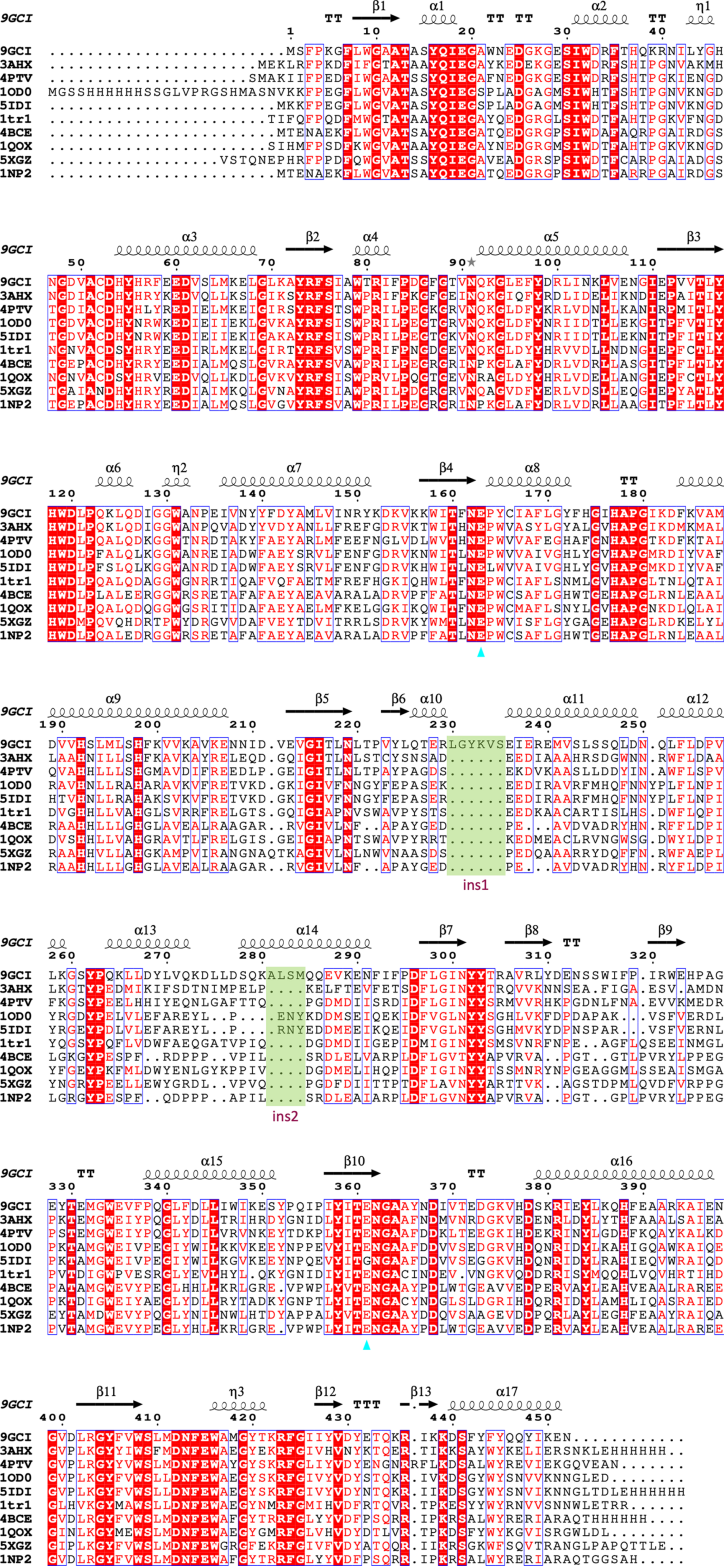
Sequence alignment of Bgl1 (PDB entry 9gci) with its closest sequence and structural homologues: β-glucosidase A from *Clostridium cellulovorans* (the closest sequence and structural homologue; PDB entry 3ahx; Jeng *et al.*, 2011 ▸), β-glucosidase from the haemophilic *Halothermothrix orenii* strain H 168 (PDB entry 4ptv; Hassan *et al.*, 2015 ▸), β-glucosidase from *Thermotoga maritima* (PDB entry 1od0; Zechel *et al.*, 2003 ▸), β-glucosidase from *T. neapolitana* (PDB entry 5idi; Kulkarni *et al.*, 2017 ▸), β-glucosidase from *Paenibacillus polymyxa* (PDB entry 1tr1; Sanz-Aparicio *et al.*, 1998 ▸), β-glucosidase from *Thermus thermophilus* strain HB8 (PDB entry 4bce; Teze *et al.*, 2014 ▸), β-glucosidase from *Niallia circulans* subsp. *alkalophilus* (PDB entry 1qox; Hakulinen *et al.*, 2000 ▸), a metagenomic glucose-tolerant β-glucosidase (PDB entry 5xgz; Matsuzawa *et al.*, 2017 ▸) and β-glucosidase from *Thermus nonproteolyticus* (PDB entry 1np2; Wang *et al.*, 2003 ▸). Identical and similar residues are shown in white on a red background and in red on a white background, respectively. The residues of interest (cyan triangles) are also indicated on the same line. The secondary-structure elements are shown for the Bgl1 structure, with α-helices, 3_10_-helices, β-strands and β-turns being denoted α, η, β and TT, respectively. The shaded regions labelled ins1 and ins2 depict the two insertions in the sequence of Bgl1. The sequence identity, coverage and *Z*-score for the closest structural homologues are summarized in Table 2 ▸.

Sequence analysis was performed using the *Basic Local Alignment Search Tool* (*BLAST*) from the National Centre for Biotechnology Information (NCBI; Altschul *et al.*, 1997 ▸). A multiple sequence alignment of homologous enzymes was performed with *Clustal Omega* on the EBI server (https://www.ebi.ac.uk/Tools/msa/clustalo/; Madeira *et al.*, 2022 ▸) and the results were visualized using *ESPript* 3.0 (https://espript.ibcp.fr; Robert & Gouet, 2014 ▸; Fig. 2 ▸) for the enzymes that had the highest structural similarity to Bgl1. Assignment of the secondary structure and analysis were performed with *PROMOTIF* as implemented in *PDBsum* (https://www.ebi.ac.uk/thornton-srv/databases/pdbsum/Generate.html; Laskowski *et al.*, 1993 ▸, 2018 ▸) on the EBI server (Supplementary Figs. S1 and S2 and Table S1).

### 2.4. Crystallization and X-ray data collection

Purified Bgl1 was concentrated to 16.7 mg ml^−1^ in 20 m*M* Tris–HCl pH 6.0 buffer and crystallization trials were performed using the sitting-drop vapour-diffusion method. A large number of conditions were explored in 96-well MRC 2 Well Crystallization Plates – UVXPO (Jena Bioscience, Cambridge, United Kingdom) with the aid of an OryxNano crystallization robot (Douglas Instruments, Hungerford, United Kingdom) installed at INSTRUCT-EL Hub/National Hellenic Research Foundation (NHRF) using commercially available crystallization screens. The final drop volumes were 500 nl and different protein:reservoir mixing ratios were explored. Plates were incubated at 19°C and crystal growth was monitored *via* a Rock Imager automated imaging system for protein crystallization (Formulatrix, USA) also installed at INSTRUCT-EL Hub/NHRF, which captures high-resolution images at selected time intervals. Crystals of Bgl1 grew as thin plates within six days in the presence of 0.2 *M* magnesium chloride hexahydrate, 0.1 *M* bis-Tris pH 5.5, 25%(*w*/*v*) PEG 3350. Co-crystallization trials of Bgl1, under the same conditions, in the presence of different disaccharides, including lactose and cellobiose at concentrations ranging from 0.3 to 1.2 m*M* and an enzyme:substrate ratio of 1:1.5, were carried out. Co-crystals were obtained for Bgl1 in the presence of 0.6 m*M* lactose [d(+)-lactose 1-hydrate, purchased from PanReac Applichem GmbH], and in the presence of 1.2 m*M* cellobiose. The Bgl1 crystals and co-crystals were flash-cooled to 100 K in a nitrogen stream using 30% glycerol as a cryoprotectant. The crystals were exposed to X-rays for 0.04 s at 100 K and diffraction data were collected to 1.41 and 1.8 Å for Bgl1 and its complex, respectively, on beamline P13 at the PETRA III synchrotron-radiation source at EMBL Hamburg (λ = 0.9763 Å, Dectris PILATUS 12M detector, oscillation range 0.1°, 1300 images in total for Bgl1 and 3600 for its complex). Data processing was performed with *XDS* (Kabsch, 2010 ▸) followed by data integration and scaling with *AIMLESS* (Evans, 2011 ▸; Evans & Murshudov, 2013 ▸) as implemented in the *CCP*4 program suite (Agirre *et al.*, 2023 ▸). The resolution cutoff was set to 1.47 and 1.95 Å for the native and complex structures, respectively, applying the criteria described by Karplus & Diederichs (2015 ▸). X-ray diffraction data analysis showed that although complete high-resolution data sets were collected from both Bgl1 crystals and co-crystals, it was only the crystals that formed in the presence of lactose that showed additional density at the active site of the enzyme that was sufficient to accommodate a sugar moiety, the product of the enzymatic reaction (Fig. 1 ▸). More specifically, the Bgl1 crystals grew in a primitive monoclinic lattice, belonging to space group *P*2_1_, with unit-cell parameters *a* = 68.4, *b* = 98.7, *c* = 80.5 Å, α = γ = 90, β = 97.5° and two molecules per asymmetric unit. The co-crystals of Bgl1 in the presence of lactose also grew in the same space group, with unit-cell parameters *a* = 68.2, *b* = 98.3, *c* = 81.8 Å, α = γ = 90, β = 97.6°. Data-collection statistics for Bgl1 and its complex are presented in Table 1 ▸.

### 2.5. Structure determination, refinement and analysis

**Figure 3:**
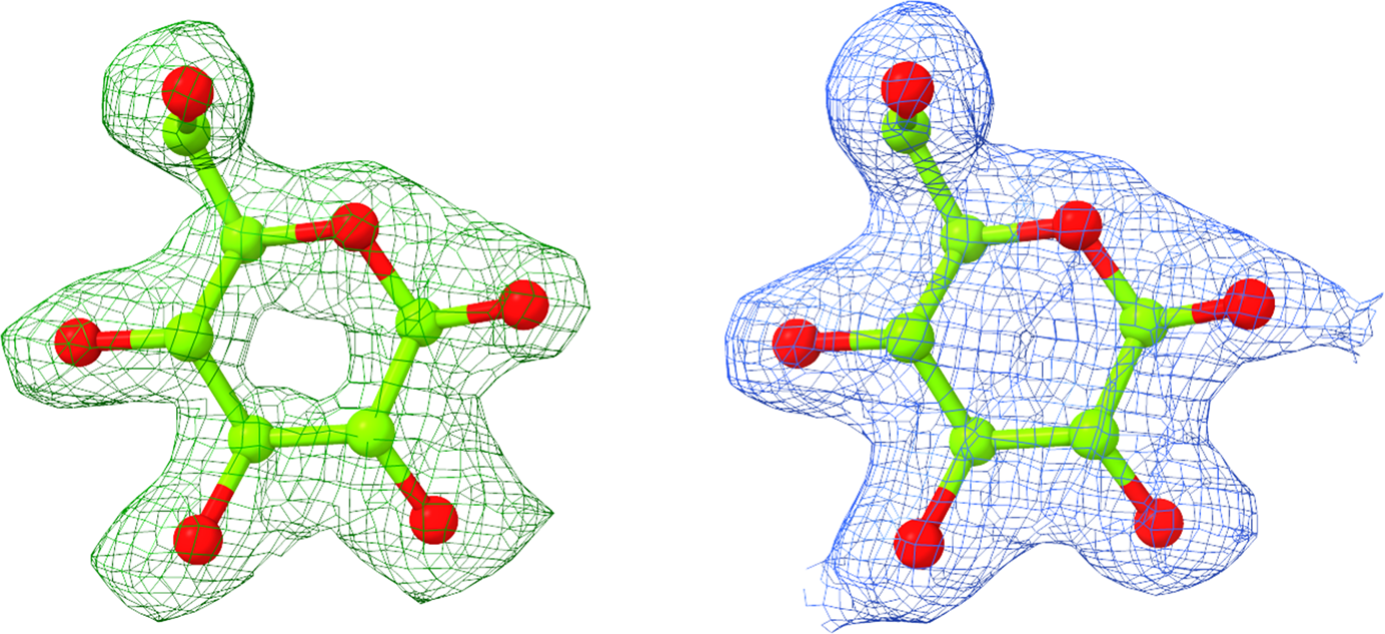
Schematic representation of the unbiased *F*_obs_ − *F*_calc_ electron-density map (green density; left) and the 2*F*_obs_ − *F*_calc_ electron-density map (blue density; right) contoured at the 3.0σ and 1.0σ levels, indicating the position of the refined β-d-glucose bound at the catalytic site of Bgl1.

The structure of Bgl1 was solved using the *BALBES* molecular-replacement pipeline (Long *et al.*, 2008 ▸) and the model generated had a 99% probability of being a solution based on the three-dimensional structure of β-glucosidase from *Clostridium cellulovarans* (PDB entry 3ahx; Jeng *et al.*, 2011 ▸), with a *Q* factor of 0.816. The model was then subjected to restrained refinement against the experimental data using *REFMAC*5 (Kovalevskiy *et al.*, 2018 ▸) from the *CCP*4 program suite (Agirre *et al.*, 2023 ▸). Alternate rounds of model building and refinement of the structure were performed using *Coot* (Emsley *et al.*, 2010 ▸) and *REFMAC*5. Solvent molecules that fulfilled the criteria of forming direct or water-mediated hydrogen-bond interactions with the protein were incorporated into the model, also using *Coot*. Visual inspection of the 2*F*_obs_ − *F*_calc_ and *F*_obs_ − *F*_calc_ electron-density maps clearly showed the binding of a glycerol (GOL) molecule at the active site of Bgl1, which was used as cryoprotectant prior to exposure of the crystals to the cryostream (Supplementary Fig. S4). It was also observed that there was sufficient density to accommodate polyethylene and ethylene glycol molecules, *i.e.* pentaethylene glycol (1PE), triethylene glycol (PGE), di­(hydroxyethyl)ether glycol (PEGL) and ethylene glycol (EDO), as well as a chlorine ion, that were present in the crystallization medium. With the aid of the difference-map peaks option implemented in *Coot*, it was observed that additional portions of density were present in the structure that could be attributed to nonspecific binding of more polyethylene glycol molecules, particularly in regions that were either exposed to the solvent or lying at the interface of the dimer with its symmetry-related molecules. However, these features were not strong enough to justify their inclusion in the model. They also induced quite a few perturbations in the vicinity of the residues located in these regions. The side chains of these residues showed that they could adopt more than one conformation, without having clear evidence from the difference maps to include them in the structure. Furthermore, extra portions of density with quite strong difference-map peaks were also detected. Selected molecules that were present during sample preparation, crystallization or X-ray data collection (for example chlorine, magnesium and water) were modelled in the density, but when the model was subjected to refinement their presence could not be justified. The structure was refined to a final *R* factor of 0.153 and a final *R*_free_ of 0.173. The refinement statistics are presented in Table 1 ▸. Similarly, the structure of Bgl1 in complex with lactose was determined by employing the Bgl1 structure as a starting model and following a standard protocol for refinement and model building as described above. Visual inspection of the 2*F*_obs_ − *F*_calc_ and *F*_obs_ − *F*_calc_ electron-density maps showed that there was sufficient density at the catalytic site of the enzyme to accommodate a glucose molecule, which was derived from the hydrolysis of lactose in the Bgl1 crystal (Fig. 3 ▸).

Additional density was also observed in the Bgl1–Glc complex for 1PE, PEG and a chlorine ion, which were included in the model as in the case of the Bgl1 structure. The same approach as followed for the Bgl1 structure was employed for the complex structure to explore the additional portions of density detected with the aid of difference-map peaks using *Coot*. The strong difference-map peaks that were observed in the Bgl1 structure were also present in the complex structure. The possibility of attributing these to known ligands based on the experimental conditions used was explored, but the results obtained after refinement could not substantiate their binding.

It is noted that in both structures additional density was observed at the N-terminus of one of the two monomers. A total of seven amino acids were modelled at the N-terminus of molecule *A* and only five at the corresponding position in molecule *A* of the Bgl1 complex. These residues originate from the plasmid section (eight amino acids, Gly-Ser-His-Met-Lys-Glu-Asp-Pro) between the thrombin cleavage site and the BamHI site in the multicloning area of the plasmid.

The diffraction precision index (DPI) was calculated using the online computing server at http://cluster.physics.iisc.ac.in/dpi/ (Kumar *et al.*, 2015 ▸; Helliwell, 2023 ▸). The stereochemistry of the protein residues was validated using *MolProbity* (Williams *et al.*, 2018 ▸). Potential hydrogen-bond and van der Waals interactions were calculated using *CONTACT* (Winn *et al.*, 2011 ▸) as implemented in the *CCP*4 program suite (Agirre *et al.*, 2023 ▸), applying distance cutoffs of 3.3 and 4.0 Å. Structural superpositions were performed with *SUPERPOSE* (Winn *et al.*, 2011 ▸) and schematic representations of all crystal structures were prepared with *UCSF ChimeraX* (Pettersen *et al.*, 2021 ▸). Structural classification of Bgl1 folding was performed using the CATH database of domain structures (Sillitoe *et al.*, 2021 ▸). The topology of the Bgl1 structure was depicted using *PDBsum* (https://www.ebi.ac.uk/; Laskowski & Thornton, 2022 ▸; Supplementary Figs. S1 and S2). Analysis of potentially identified protein cavities at the protein surface was performed with *DeepSite* (http://www.playmolecule.org; Jiménez *et al.*, 2017 ▸). Structural homologues of Bgl1 were identified using the *DALI* server (Holm *et al.*, 2023 ▸).

### 2.6. PDB accession codes

The atomic coordinates and structure factors for the crystal structures of Bgl1 and its complex with β-d-glucose have been deposited in the Protein Data Bank (https://www.pdb.org) under accession codes 9gci and 9gcj, respectively.

## 3. Results and discussion

### 3.1. Sequence analysis

A sequence-similarity search against all nonredundant sequences performed with* BLAST* showed that Bgl1 (PDB entry 9gci) shares high sequence similarity with a large number of both hypothetical and characterized proteins that belong to the GH1 family. The closest known sequence homologue is β-glucosidase A from *C. cellulovorans* (which is both the closest sequence and structural homologue; PDB entry 3ahx; Jeng *et al.*, 2011 ▸). The corresponding characterized homologues from thermoresistant organisms are a β-glucosidase from the haemophilic *Halothermothrix orenii* strain H 168 (PDB entry 4ptv; Hassan *et al.*, 2015 ▸), a β-glucosidase from *Thermotoga maritima* (PDB entry 1od0; Zechel *et al.*, 2003 ▸), a β-glucosidase from *T. neapolitana* (PDB entry 5idi; Kulkarni *et al.*, 2017 ▸), a β-glucosidase from *Paenibacillus polymyxa* (PDB entry 1tr1; Sanz-Aparicio *et al.*, 1998 ▸), a β-glucosidase from *Thermus thermophilus* strain HB8 (PDB entry 4bce; Teze *et al.*, 2014 ▸), a β-glucosidase from *Niallia circulans* subsp. *alkalophilus* (PDB entry 1qox; Hakulinen *et al.*, 2000 ▸), a metagenomic glucose-tolerant β-glucosidase (PDB entry 5xgz; Matsuzawa *et al.*, 2017 ▸) and a β-glucosidase from *Thermus nonproteolyticus* (PDB entry 1np2; Wang *et al.*, 2003 ▸) (Table 2 ▸). Furthermore, multiple sequence alignment revealed that Bgl1 has an insertion of eight amino acids (454–460) that is not present in any of the other β-glucosidases.

### 3.2. Analysis of the Bgl1 crystal structures

The Bgl1 crystals grew in the monoclinic space group *P*2_1_with two molecules per asymmetric unit. The three-dimensional structure of β-glucosidase from *C. cellulovarans* (PDB entry 3ahx) was used as a starting model. The derived model was subjected to refinement and was enriched by the insertion of residues that corresponded to the translated sequence of the pET-15b plasmid between the thrombin cleavage site and the gene-insertion point (BamHI) that was used for preparation of the recombinant protein. Validation of the final structures showed that most of the residues lay in allowed regions of the Ramachandran plot and the geometry indicators are of high quality (Table 1 ▸). Two *cis*-peptide bonds were found at Ala178–Pro179 and Trp408–Ser409. The presence of such bonds is typical of glycosyl hydrolase family 1 (Seshadri *et al.*, 2009 ▸).

The overall structures of Bgl1 and its complex exhibit the classical (β/α)_8_ TIM-barrel fold, which is common to all PDB-deposited structures of glycosyl hydrolase family 1 enzymes (GH1 superfamily; EC 3.2.1.21). Comparison of the two monomers in each individual structure showed that they have negligible differences, with an r.m.s.d. on C^α^ atoms or secondary-structure elements of 0.16 Å for Bgl1. Superposition of the molecules in the asymmetric unit of the Bgl1 complex structure onto the Bgl1 structure showed that the r.m.s.d. on C^α^ atoms (453 residues) is 0.32 Å and that on secondary-structure elements (905 residues) is 0.36 Å. The catalytic site residues of the enzyme are two glutamic acid residues, Glu163 and Glu361, as in other enzymes that belong to the same family. The catalytic acid/base Glu163 is located at the end of β-strand 4 and the catalytic nucleophile Glu361 is located at the end of β-strand 10.

**Figure 4:**
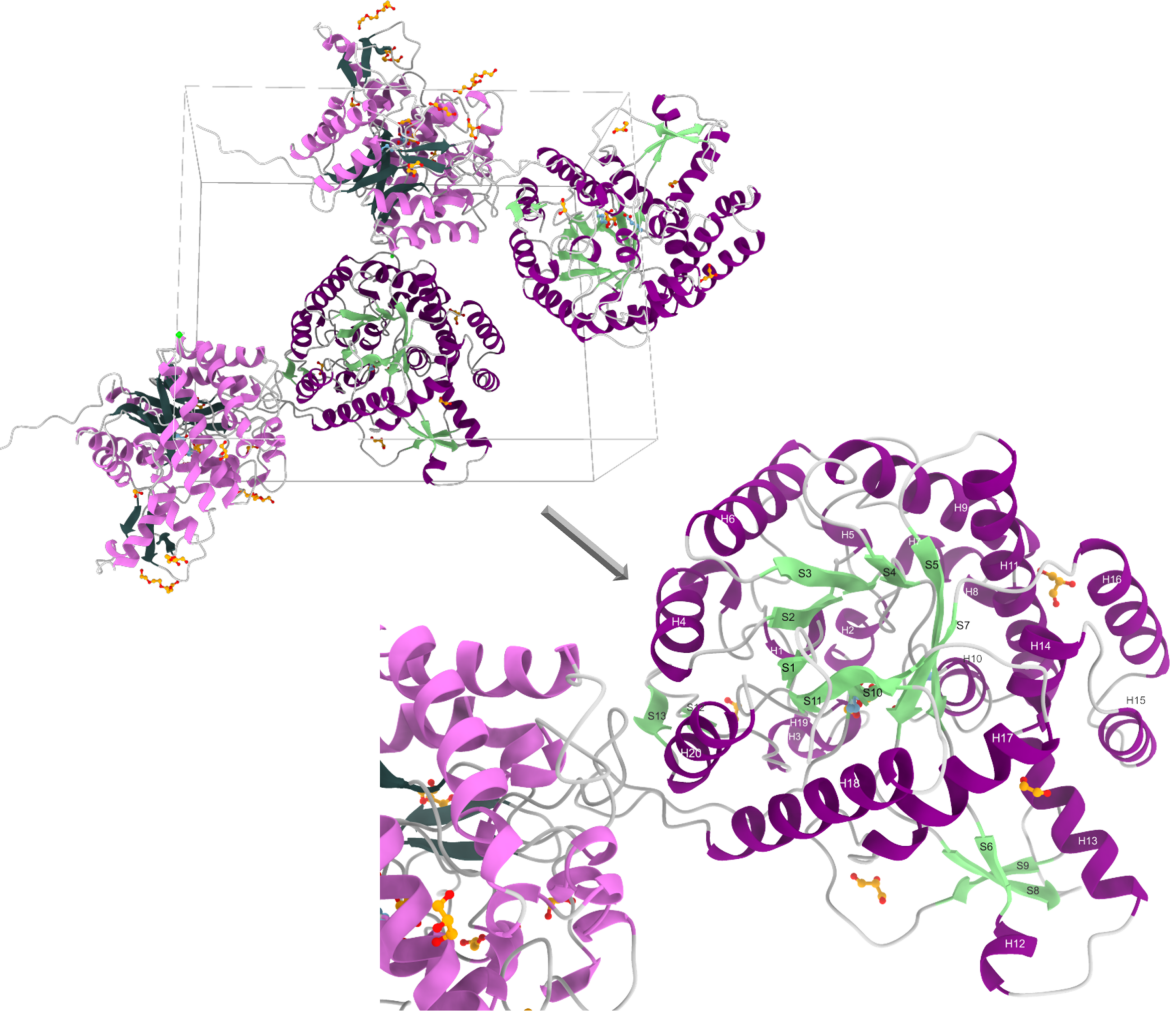
Schematic representation of the Bgl1 crystal packing. The unit cell is shown in grey and the Cl^−^ ion is indicated as a green sphere. A closer view of the (β/α)_8_ TIM-barrel fold is presented with the α-helices (shown in mauve) and β-strands (shown in lime green) labelled.

In the case of Bgl1, sufficient electron density to accommodate a glycerol (GOL) molecule and an ethylene glycol (EDO) molecule was observed at the active site of the enzyme and both were added to the model, which was then subjected to refinement. Both molecules form hydrogen bonds and van der Waals interactions with the catalytic residues Glu361 and Glu163, respectively, and mimic the binding mode of the substrate (Supplementary Tables S4–S9 and Figs. S4 and S5). An additional glycerol molecule is bound at the interface of the first monomer (chain *A*) with a symmetry-related molecule of the second monomer (chain *B*; symmetry operator −*x*, *y* + 1/2, −*z*). The 2*F*_obs_ − *F*_calc_ and *F*_obs_ − *F*_calc_ electron-density maps also revealed the binding of polyethylene glycol (PEG) molecules that lie on the surface of the Bgl1 structure in those areas which are most exposed to the solvent and are not involved in symmetry-related packing interactions. One chlorine ion (Cl^−^) was also included in the structure, as suggested by both the 2*F*_obs_ − *F*_calc_ and *F*_obs_ − *F*_calc_ electron-density maps; it was bound at the interface formed between chain *A* and the symmetry-related molecule of chain *B* (Fig. 4 ▸, Supplementary Fig. S3 and Tables S2 and S3). The presence of the aforementioned molecules in the structure is attributed to the crystallization medium and/or the cryoprotectants used, which explains their binding; however, sufficient evidence is not provided that any of these molecules may have specificity for any of these sites.

In the case of the Bgl1 complex, the overall structure is the same as that of Bgl1. A β-d-glucose molecule is bound at the active site of the enzyme, as clearly indicated by additional density present at the same position at which GOL bound in the free form (Figs. 3 ▸ and 5 ▸). Comparison of these two structures showed that the residues lining the catalytic site of the enzyme have the same conformation, except for the side chain of Glu163, the χ_3_ torsion angle of which rotates by ∼30°, and a negligible change of Asn219, the χ_1_ torsion angle of which rotates by ∼15° to optimize the interaction with glucose. The crystal structure of the enzyme also includes a chlorine ion in the same position as in the Bgl1 structure and a total of two molecules of 1PE (also present as PEG in Bgl1) and one molecule of PEG (bound very close to the position previously occupied by glycerol in Bgl1), as indicated by the 2*F*_obs_ − *F*_calc_ and *F*_obs_ − *F*_calc_ electron-density maps. The rest of the differences observed in the vicinity of the active site may be attributed to the glycerol molecule entrapped between the two symmetry-related molecules in Bgl1 that induces changes to the side chain of His324, the torsion angles (χ_1_ and χ_2_) of which rotate by ∼78° and 34.1°, respectively, in the presence of GOL504. This change is additionally associated with a change in the side chain of Trp322, which is also subjected to a rotation of the χ_2_ torsion angle by 131.4°. These two changes induce a plausible perturbation in the solvent that is also reflected in residue Asp249, the side chain of which rotates by ∼43° (χ_2_). The nonspecific binding of polyethylene glycol molecules also introduces some local disturbance, but this is not sufficiently significant to correlate with a functional role of the enzyme.

### 3.3. Comparison of Bgl1 with β-glucosidase A from *C. cellulovorans* and other structural homologues

β-Glucosidase from *C. cellulovarans* (PDB entry 3ahx; Jeng *et al.*, 2011 ▸) is the closest sequence and structural homologue of Bgl1, with an r.m.s.d. of 1.1 Å as calculated by the *DALI* server (Holm *et al.*, 2023 ▸). The overall structures of the two proteins are quite similar. The catalytic site residues remain unchanged in both structures; however, the residues in the vicinity of the catalytic residues exhibit differences. The constellation of several residues neighbouring Glu163, comprising Tyr165, Cys166, Phe169, Leu248 and Trp316, replace the corresponding residues Trp, Val, Tyr, Trp and Phe in the structure of β-glucosidase from *C. cellulovarans* and induce local changes that may be attributed to the charge and volume of their side chains. In particular, Tyr165 is only present in Bgl1, while in all of the other structural homologues a tryptophan is present (Fig. 2 ▸). These changes are reflected in residues 309–328 lining strands β8 and β9 and the loop region that connects them (*i.e.* residues 312–319), the C^α^ atoms of which are subjected to shifts of up to ∼4.2 Å in the loop.

**Figure 6:**
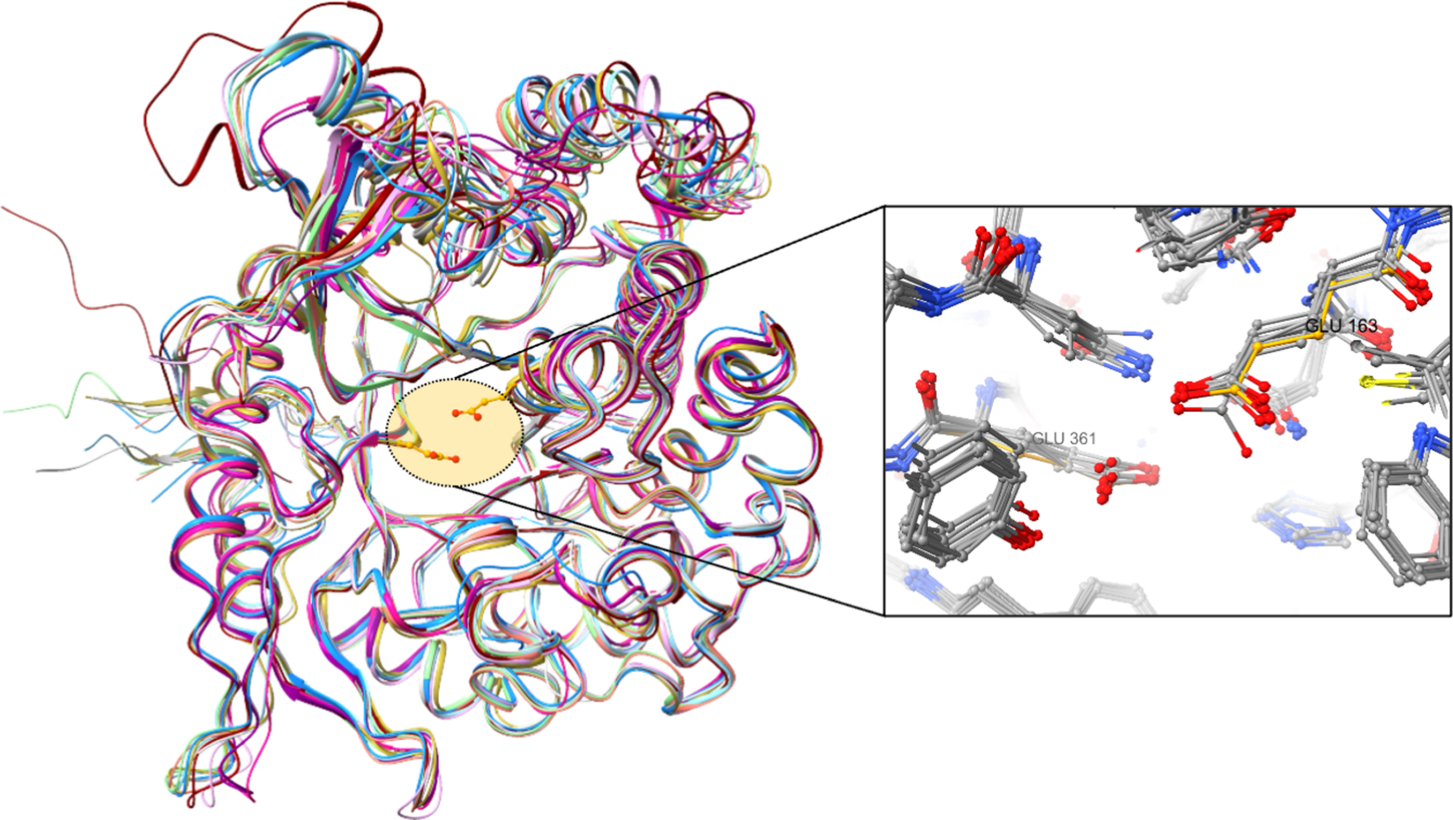
Left: superposition of the crystal structures of Bgl1 (shown in dark red) with its closest sequence and structural homologues β-glucosidase A from *Clostridium cellulovorans* (PDB entry 3ahx; cyan; Jeng *et al.*, 2011 ▸), β-glucosidase from haemophilic *Halothermothrix orenii* strain H 168 (PDB entry 4ptv; pink; Hassan *et al.*, 2015 ▸), β-glucosidase from *Thermotoga maritima* (PDB entry 1od0; light green; Zechel *et al.*, 2003 ▸), β-glucosidase from *T. neapolitana* (PDB entry 5idi; orange; Kulkarni *et al.*, 2017 ▸), β-glucosidase from *Paenibacillus polymyxa* (PDB entry 1tr1; grey; Sanz-Aparicio *et al.*, 1998 ▸), β-glucosidase from *Thermus thermophilus* strain HB8 (PDB entry 4bce; magenta; Teze *et al.*, 2014 ▸), β-glucosidase from *Niallia circulans* subsp. *alkalophilus* (PDB entry 1qox; khaki; Hakulinen *et al.*, 2000 ▸), a metagenomic glucose-tolerant β-glucosidase (PDB entry 5xgz; blue; Matsuzawa *et al.*, 2017 ▸) and β-glucosidase from *Thermus nonproteolyticus* (PDB entry 1np2; mauve; Wang *et al.*, 2003 ▸). Right: close-up view of the active site indicating the catalytic site residues Glu163 and Glu361 (the numbering is from the Bgl1 structure).

Sequence alignment of the structural homologues of Bgl1 (Fig. 2 ▸) showed that there are two insertions that affect the structure of Bgl1. The first is observed in the loop region (residues 230–235) that connects 3_10_-helix α10 and helix α11 and is unique to Bgl1, since it does not appear in any of its structural homologues. The second insertion involves part of helix α14, which is more extended (residues 281–284) compared with the corresponding helix in *C. cellulovorans*, for example. It is only the β-glucosidases from *Thermotoga maritima* (PDB entry 1odo) and *T. neapolitana* (PDB entry 5idi) that have a similar insertion, although with a different sequence. Further comparison with the *C. cellulovorans* β-glucosidase structure reveals additional changes in the loop region that connects α9 and β5 (C^α^-atom shifts vary from ∼0.9 to ∼3.5 Å, with the most profound shifts at Glu207 and Asp211). These changes may be attributed to the lack of aromatic side chains of Ile149 and Val205 in Bgl1 compared wth Phe and Tyr, leading to a more compact structure in the region (Fig. 6 ▸).

**Figure 7:**
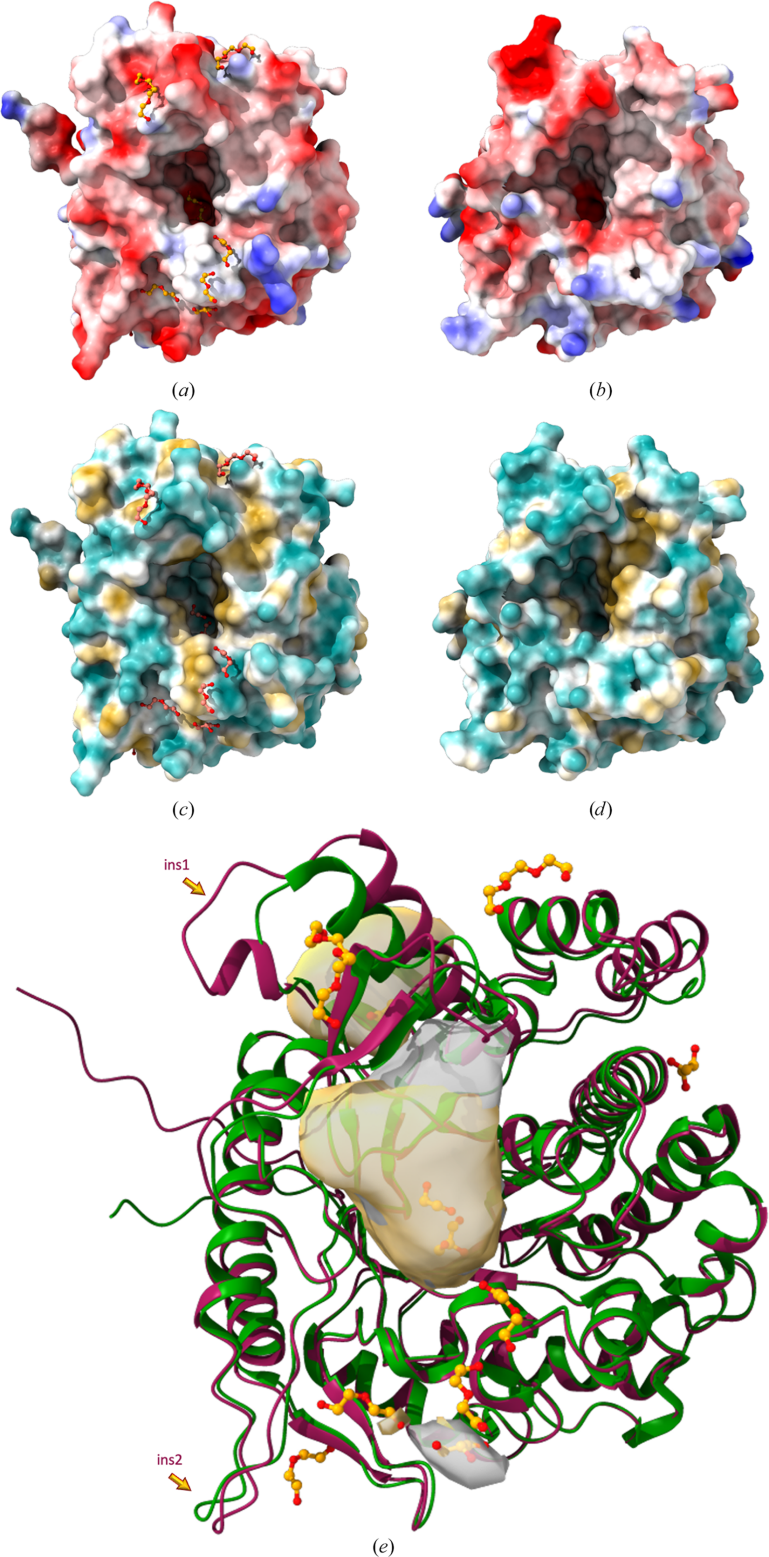
(*a*, *b*) Electrostatic (Coulomb) potential surface representations of (*a*) Bgl1 from *C. saccharolyticus* and (*b*) β-glucosidase from *C. cellulovorans* (PDB entry 3ahx); the colouring ranges from red for negative potential through white to blue for positive potential. (*c*, *d*) Hydrophobic surface representation of (*c*) Bgl1, also indicating the position of the bound (poly)ethylene glycol and glycerol molecules, and (*d*) β-glucosidase from *C. cellulovorans* (PDB entry 3ahx); the colouring ranges from dark cyan (most hydrophilic) to white to dark yellow (most lipophilic). (*e*) Superposition of the crystal structures of Bgl1 (red) and β-glucosidase from *C. cellulovorans* (PDB entry 3ahx; green); the regions in the two structures that exhibit the most profound differences are indicated with arrows (see Fig. 2 ▸ for the corresponding residues). The catalytic site of Bgl1 and an additional cavity adjacent to it that was detected by *DeepSite* (Jiménez *et al.*, 2017 ▸) are highlighted in surface representation (yellow).

With the aim of further investigating the insertions present in Bgl1, *DeepSite* (http://www.playmolecule.org), a machine-learning algorithm based on deep convolutional neural networks (DCNNs) for detecting druggable binding sites in proteins targeted for structure-based drug design, was used. The algorithm can identify and define protein cavities, potentially at the protein surface, that are likely to bind a small compound. *DeepSite* (Jiménez *et al.*, 2017 ▸) was used for both the Bgl1 structure and the *C. cellulovorans* β-glucosidase structure and the results are depicted in Fig. 7 ▸. The results showed that there is a distinct cavity that *DeepSite* identified in the Bgl1 structure adjacent to the catalytic site, the formation of which was possible due to the presence of the additional residues (Fig. 7 ▸, ins1, surface shown in yellow). This observation paves the way for further investigations involving protein-engineering studies that will allow the elucidation of the functional role of this region. The cavity identified may serve as a region that fosters binding of the substrate, navigating it to the active site and increasing the catalytic efficiency of the enzyme.

## 4. Conclusions

In the present study, we report the structural characterization of Bgl1, a thermostable enzyme with an attractive catalytic profile for various industrial applications. The crystal structures of Bgl1 from the thermophilic bacterium *C. saccharo­lyticus* and its complex with glucose were determined at 1.47 and 1.95 Å resolution, respectively. Bgl1 is a member of glycosyl hydrolase family 1 (GH1 superfamily, EC 3.2.1.21), the members of which have a classical (β/α)_8_ TIM-barrel fold. The results showed that the 3D structure of Bgl1 from *C. saccharolyticus* follows the overall architecture of the GH1 family. Calculation of 2*F*_obs_ − *F*_calc_ and *F*_obs_ − *F*_calc_ electron-density maps showed that at the catalytic site of the enzyme, a glycerol molecule was bound to Bgl1 and interacted with the catalytic residues Glu163 and Glu361, but when lactose was used as substrate analogue, β-d-glucose, the product of the catalytic reaction taking place in the crystal, was bound at the same site. Comparison of Bgl1 with sequence or structural homologues of β-glucosidase showed that Bgl1 is quite similar except for two regions, one of which seems to be a unique insertion of residues that is only present in Bgl1 (Fig. 7 ▸*e*). This region comprises a flexible loop and adopts a different conformation compared with other enzymes, as becomes evident from superposition of their overall structures based on secondary-structure elements. The formation of an additional cavity adjacent to the catalytic site of Bgl1 was identified using *DeepSite*, a new tool that uses deep convolutional neural networks to detect potential binding sites. The importance of this insertion for the catalytic efficiency of Bgl1 has yet to be elucidated through protein engineering to decipher the plausible functional role of this region.

## 5. Related literature

The following reference is cited in the supporting information for this article: Hutchinson & Thornton (1996 ▸).

## Supplementary Material

PDB reference: β-glucosidase from *Caldicellulosiruptor saccharolyticus*, 9gci

PDB reference: complex with β-d-glucose, 9gcj

Supplementary Tables and Figures,. DOI: 10.1107/S2059798324009252/gm5108sup1.pdf

## Figures and Tables

**Figure 5:**
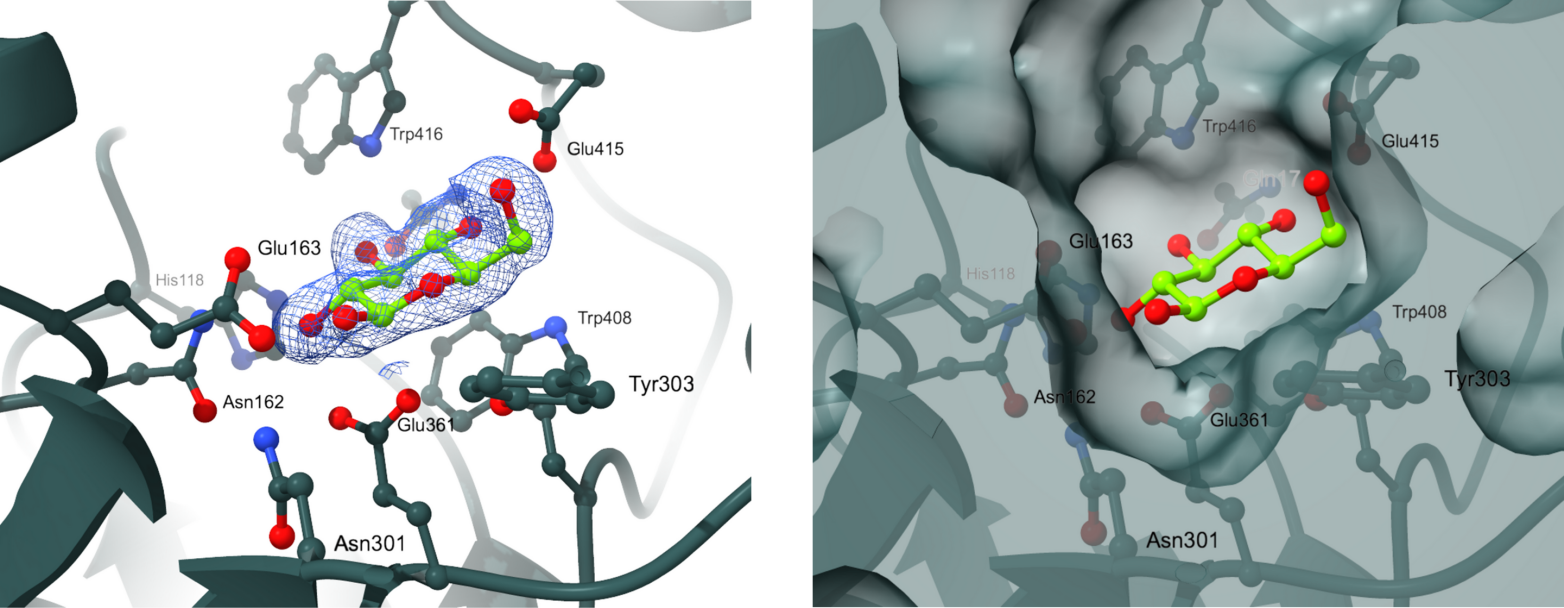
Schematic representation of β-d-glucose bound at the catalytic site of the enzyme.

**Table 1:** Diffraction data and refinement statistics for Bgl1 and Bgl1 in complex with glucose Values in parentheses are for the outermost shell.

	Bgl1 (PDB entry 9gci)	Bgl1–glucose (PDB entry 9gcj)
Data-collection and processing statistics		
Crystallization conditions	0.2 *M* magnesium chloride hexahydrate, 0.1 *M* bis-Tris pH 5.5, 25%(*w*/*v*) PEG 3350	0.2 *M* magnesium chloride hexahydrate, 0.1 *M* bis-Tris pH 5.5, 25%(*w*/*v*) PEG 3350, 0.6 m*M* lactose
Source	Beamline P13, PETRA III	Beamline P13, PETRA III
Wavelength (Å)	0.9763	0.9763
Space group	*P*2_1_	*P*2_1_
*a*, *b*, *c* (Å)	68.4, 98.7, 80.5	68.2, 98.3, 81.8
α, β, γ (°)	90, 97.5, 90	90, 97.6, 90
No. of molecules per asymmetric unit	2	2
Resolution (Å)	67.86–1.47 (1.50–1.47)	98.31–1.95 (1.99–1.95)
No. of observations	438858 (21514)	519537 (30744)
No. of unique reflections	173397 (8680)	77506 (4600)
Completeness (%)	96.5 (97.7)	99.6 (99.9)
*R*_meas_†	0.068 (0.627)	0.137 (1.655)
*R*_p.i.m._†	0.060 (0.574)	0.095 (0.634)
〈*I*/σ(*I*)〉†	8.6 (1.6)	8.3 (1.8)
CC_1/2_†	0.997 (0.668)	0.974 (0.653)
Multiplicity	2.5 (2.5)	6.7 (6.7)
Wilson *B* value (Å^2^)	10	25
Refinement statistics and model quality		
No. of reflections (all/free)	173368/8764	77479/3968
Residues included		
Chain *A*	460	458
Chain *B*	452	453
No. of protein atoms	7605	7592
No. of heteroatoms		
Solvent molecules	829	402
Ions	1 Cl^−^	1 Cl^−^
Polyethylene glycol‡	13 1PE, 10 PGE, 35 PEGL	32 1PE, 7 PEGL
Ethylene glycol	16	—
Glycerol	36	—
β-D-Glucose	—	Chain *A*, 12; chain *B*, 12
*R*/*R*_free_§	0.153/0.173	0.171/0.207
R.m.s. deviations		
Bond lengths (Å)	0.0053	0.0050
Bond angles (°)	1.3	1.2
*MolProbity* analyses¶		
Ramachandran outliers (%)	0.2	0.0
Ramachandran favoured (%)	98.1	97.7
Poor rotamer outliers (%)	0.0	0.12
Average *B*, protein atoms (Å^2^)		
Overall	14	33
Backbone atoms	13	31
Side-chain atoms	16	35
Average *B*, heteroatoms (Å^2^)		
Solvent molecules	27	36
Ions	14	30
Polyethylene glycol	1PE, 33; PGE, 46; PEGL, 38	1PE, 49; PEGL, 53
Ethylene glycol	29	—
Glycerol	24	—
β-D-Glucose	—	Chain *A*, 27; chain *B*, 32
DPI††	0.058	0.148

† Indicators for assessing the collected data quality as described in Karplus & Diederichs (2015 ▸).

‡ Polyethylene glycol molecules include 1PE (pentaethylene glycol), PGE (triethylene glycol) and PEGL [di(hydroxyethyl)ether].

§ Crystallographic *R* = 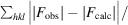

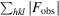
, where |*F*_obs_| and |*F*_calc_| are the observed and calculated structure-factor amplitudes, respectively. *R*_free_ is the corresponding *R* value for a randomly chosen 5% of the reflections that were not included in the refinement.

¶ Calculated using *MolProbity* (https://molprobity.biochem.duke.edu/).

†† Calculated using the online DPI computing server at http://cluster.physics.iisc.ac.in/dpi/ (Kumar *et al.*, 2015 ▸; Helliwell, 2023 ▸).

**Table 2:** The closest homologues of Bgl1 for which structures have been determined by X-ray crystallography

PDB code	Source	Sequence identity (%)	Sequence coverage (%)	*Z*-score (%)
3ahx	*Clostridium cellulovorans*	53.0	100	61.5
4ptv	*Halothermothrix orenii* strain H 168	50.1	99	59.9
1od0	*Thermotoga maritima*	47.8	99	58.2
5idi	*Thermotoga neapolitana*	47.5	99	47.0
1tr1	*Paenibacillus polymyxa*	43.6	99	57.6
4bce	*Thermus thermophilus* strain HB8	45.1	98	57.6
1qox	*Niallia circulans* subsp. *alkalophilus*	45.2	99	57.5
5xgz	Soil metagenome	44.1	99	57.4
1np2	*Thermus nonproteolyticus*	46.3	99	57.1
