# Supplementary material for: Structural studies of β-glucosidase from the thermophilic bacterium Caldicellulosiruptor saccharolyticus
Source: Acta Crystallogr D Struct Biol. 2024 Oct 1;80(Pt 10):733–43. doi: 10.1107/S2059798324009252 (PMC11448918; doi:10.1107/S2059798324009252)
Supplement: Supplementary file 1 [file d-80-00733-sup1.pdf]

STRUCTURAL BIOLOGY

Volume 80 (2024)

Supporting information for article:

Structural studies of β-glucosidase from the thermophilic bacterium *Caldicellulosiruptor saccharolyticus*

Anastasia I. Sotiropoulou, Dimitris G. Hatzinikolaou and Evangelia D. Chrysina

S1. Structural summary of Bgl1

The secondary structure elements and the topology of Bgl1 are presented in figures S1 and S2.

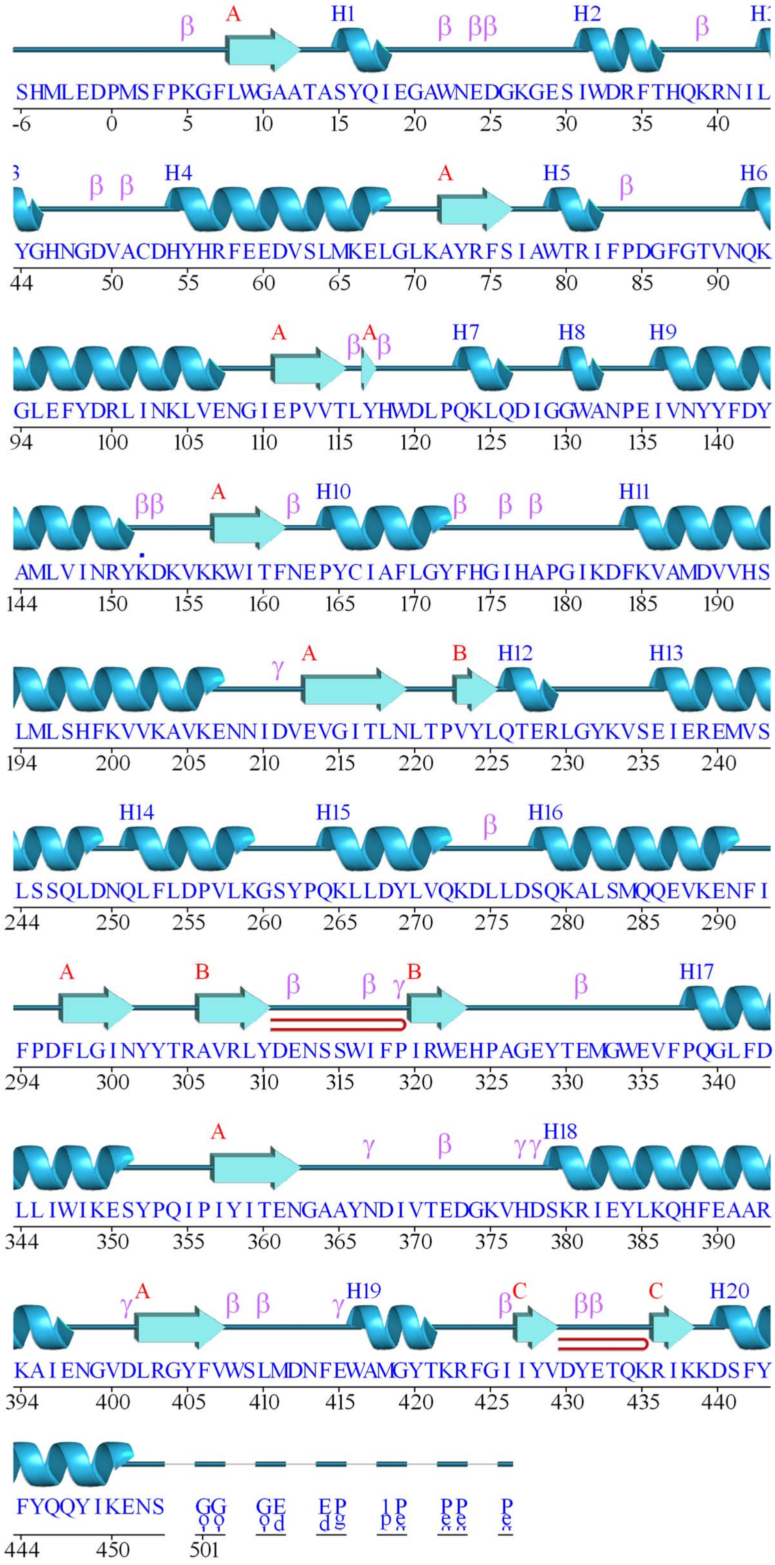

Figure S1 Schematic “wiring diagram” of the Bgl1’s secondary structure—including strands (cyan arrows), helices (light blue springs), and other motifs in red (e.g., β-hairpins, γ-turns, etc)

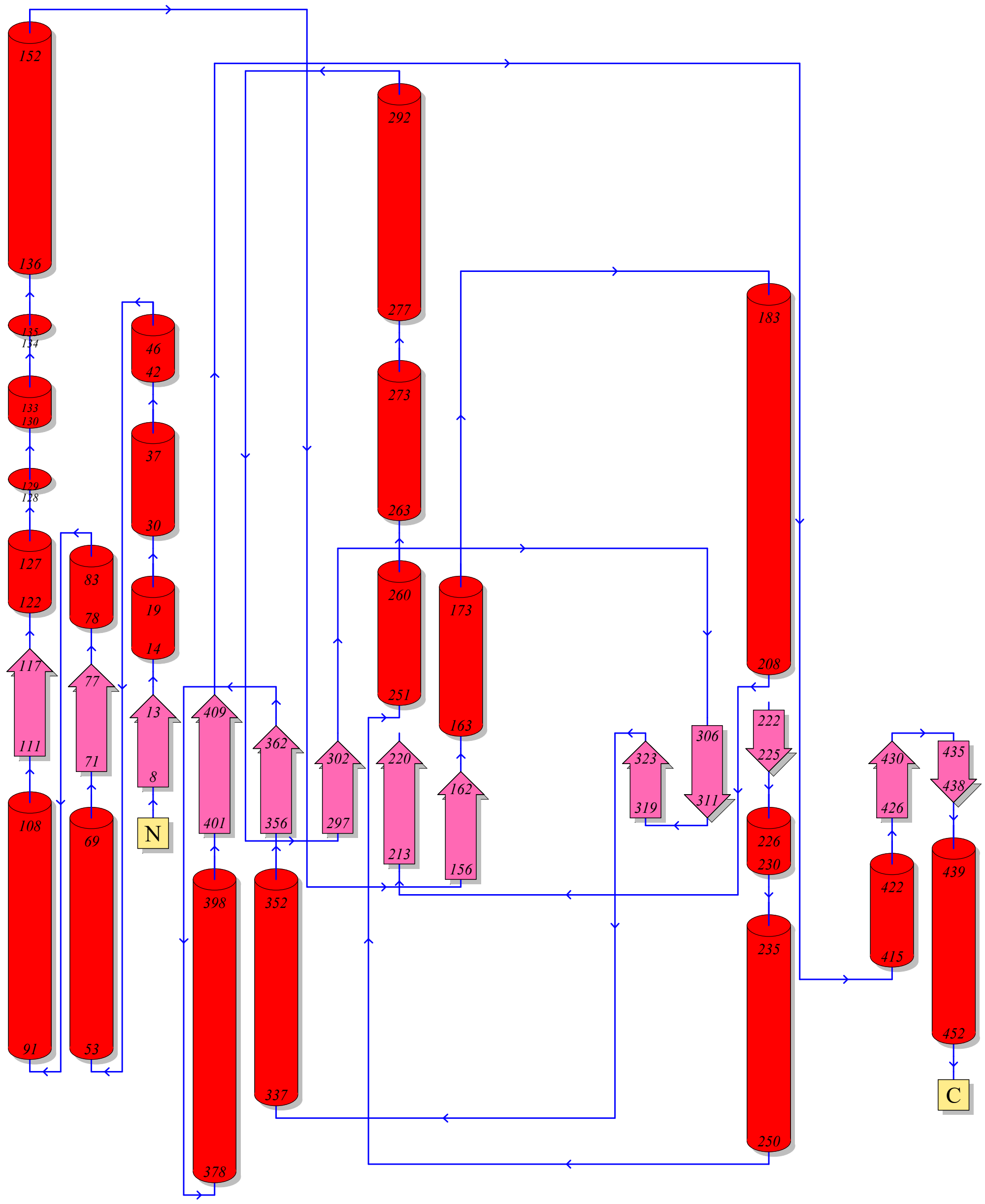

Figure S2 Schematic diagram illustrating Bgl1's topology in terms of how the β-strands (pink arrows) are arranged into β-sheets, and the relative arrangement of the α-helices (red cylinders).

Analysis of the Bgl1 secondary structure assignment according to *ProMotif* (Hutchinson & Thornton, 1996) is presented in Table S1.

Table S1 Secondary structure assignment of Bgl1 by *ProMotif* (Hutchinson & Thornton, 1996)

**β-sheets**	3
**β-α-β motifs**	5
**β-hairpins**	2
**β-bulges**	6
**Strands**	13
**Helices**	20
**Helix-helix interactions**	33
**β-turns**	26
**γ-turns**	7

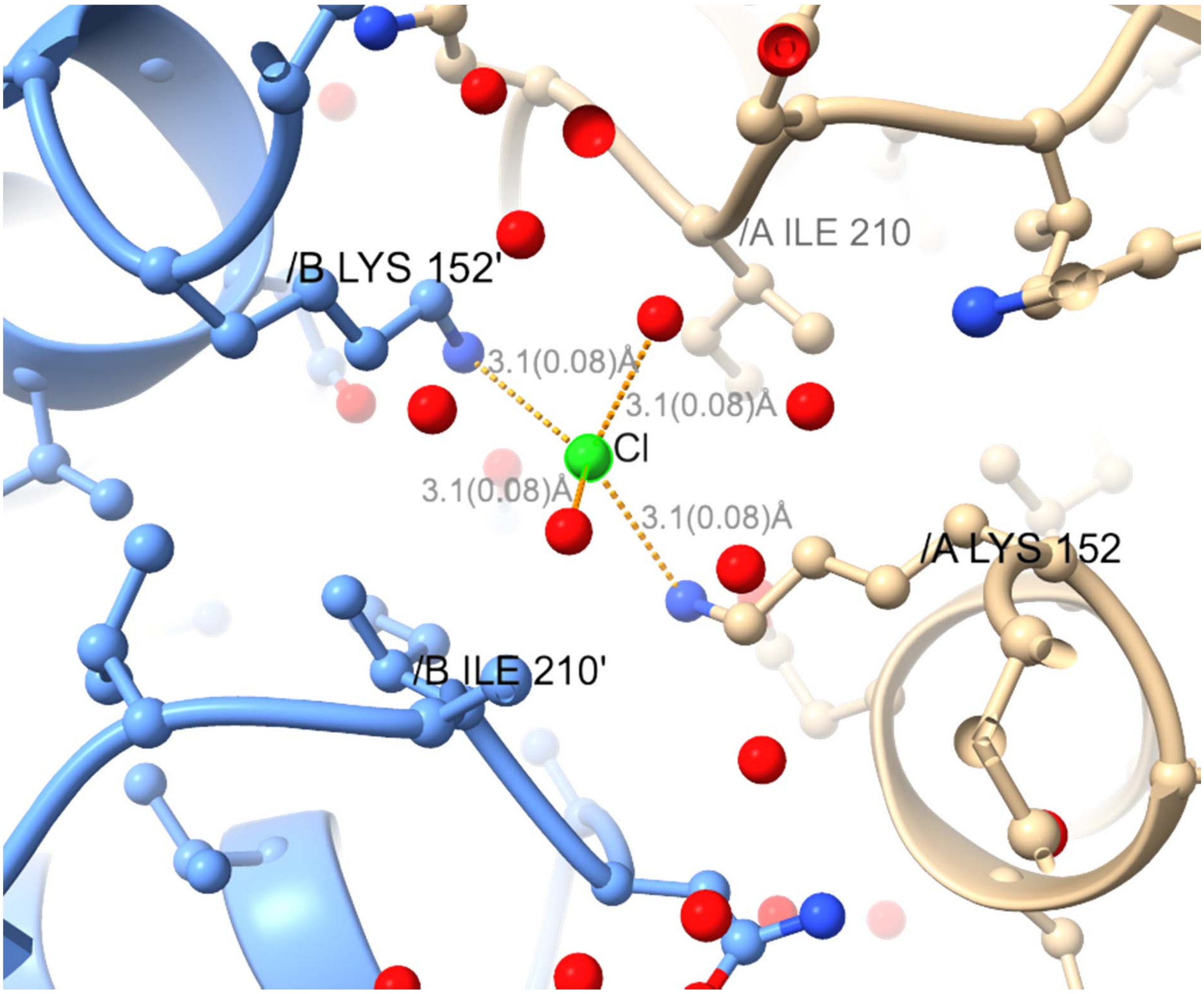

Figure S3 Schematic representation of the interactions formed by the chlorine ion bound at the crystal packing interface of molecule A and the symmetry related molecule of chain B.

Table S2 Van der Waals interactions of Cl^- in monomer A of BGl1

Atom	Protein Atom	Distance (Å)[a]
Cl	Lys152A CD	3.9 (0.08)
	Lys152A CE	3.6 (0.08)
	Wat343C O	2.1 (0.09)
	Lys152A NZ	3.0 (0.08)
	**Total 4**	

[a] The numbers in parentheses show the uncertainty standard values of the distance. The uncertainty values were calculated taking into consideration the individual uncertainty values (coordinate error) of each atom involved in the interaction, based on DPI (Kumar *et al.*, 2015; Helliwell, 2023)

Table S3 Van der Waals interactions of Cl^- in the symmetry related molecule of BGl1

Atom	Protein Atom	Distance (Å)[a]
Cl	Wat67' O	3.1 (0.08)
	Lys152B' CE	3.6 (0.07)
	Lys152B' NZ	3.1 (0.08)
	Lys152B' CD	3.9 (0.07)
	**Total 4**	

[a] The numbers in parentheses show the uncertainty standard values of the distance. The uncertainty values were calculated taking into consideration the individual uncertainty values (coordinate error) of each atom involved in the interaction, based on DPI (Kumar *et al.*, 2015; Helliwell, 2023)

Table S4 Direct hydrogen bonds and water-mediated hydrogen-bond interactions of GOL of monomer A in the catalytic site of BGl**1**

Atom	Protein Atom	Distance (Å)[a]	Angle (°)
Gol 501A O1	Glu 361A OE2	2.6 (0.07)	104.1
	Gol 501A O2	2.9 (0.07)	0.0
	Asn162A ND2	2.8 (0.07)	147.7
Gol 501A O2	Gol 501A O3	3.0 (0.08)	0.0
	Gln17A OE1	2.6 (0.07)	126.6
	His118A NE2	2.9 (0.07)	157.3
	Trp416A NE1	2.9 (0.07)	164.3
Gol 501A O3	Glu415A OE2	2.8 (0.08)	115.3
	Edo505A O2	2.9 (0.10)	0.0
		**Total 9**	

[a] The numbers in parentheses show the uncertainty standard values of the distance. The uncertainty values were calculated taking into consideration the individual uncertainty values (coordinate error) of each atom involved in the interaction, based on DPI (Kumar *et al.*, 2015; Helliwell, 2023)

Table S5 Van der Waals Interactions of GOL of monomer A in the catalytic site of BGl1

Atom	Protein Atom	Distance (Å)[a]
Gol 501A C1	Glu361A CD	3.7 (0.07)
	Glu361A OE1	3.0 (0.07)
	Gol501A O3	2.8 (0.08)
	Edo505A O2	3.3 (0.10)
	His118A NE2	4.0 (0.07)
	Gol501A O2	2.4 (0.07)
	Gol501A C3	2.5 (0.08)
	Glu163A CD	3.7 (0.07)
	Glu163A OE1	3.5 (0.07)
	Glu163A OE2	3.5 (0.07)
	Asn162A ND2	3.9 (0.07)
Gol501A O1	Glu361A CD	3.2 (0.07)
	Glu361A OE1	3.0 (0.07)
	His118A NE2	3.3 (0.07)
	Gol501A C2	2.4 (0.08)
	Gol501A C3	3.8 (0.08)
	Glu163A CG	4.0 (0.07)
	GLu163A CD	3.4 (0.07)
	Glu163A OE1	3.6 (0.07)
	Glu163A OE2	3.4 (0.07)
	Asn162A CG	3.5 (0.07)
	Asn162A OD1	3.4 (0.07)
	Asn301A ND2	3.6 (0.07)
	His118A CE1	3.5 (0.07)
Gol501A C2	Glu361A CD	3.9 (0.07)
	Glu361A OE1	3.3 (0.07)
	Glu361A OE2	3.7 (0.07)
	Trp408A CE2	3.9 (0.07)
	Gol501A O3	2.4 (0.08)
	Gln17A O3	3.7 (0.07)
	His118A NE2	3.9 (0.07)
	Trp408A CZ2	3.7 (0.07)
	Trp408A CH2	3.9 (0.07)
	Trp416A NE1	3.9 (0.07)
Gol501A O2	Gln17A CD	3.5 (0.07)
	Gln17A NE2	3.8 (0.07)
	Trp408A CZ2	3.7 (0.07)

	Trp408A CH2	3.7 (0.07)
	Trp416A CE2	3.7 (0.07)
	Trp416A CZ2	3.9 (0.07)
	Gol501A C3	2.4 (0.08)
	His118A CD2	4.0 (0.07)
	His118A CE1	3.8 (0.07)
Gol501A C3	Trp408A NE1	3.8 (0.07)
	Glu415A CD	4.0 (0.08)
	Glu415A OE2	3.6 (0.08)
	Glu415A OE1	3.5 (0.08)
	Glu361A OE1	4.0 (0.08)
	Trp408A CE2	3.7 (0.07)
	Edo505A O2	4.0 (0.10)
	Trp408A CZ2	3.7 (0.07)
	Trp416A NE1	3.9 (0.07)
Gol501A O3	Glu415A CD	3.5 (0.08)
	Glu415A OE1	3.4 (0.08)
	Edo505A C2	3.6 (0.10)
	Trp416A CD1	3.5 (0.07)
	Edo505A C2	2.9 (0.10)
	Trp416A NE1	3.5 (0.07)
	**Total 58**	

[a] The numbers in parentheses show the uncertainty standard values of the distance. The uncertainty values were calculated taking into consideration the individual uncertainty values (coordinate error) of each atom involved in the interaction, based on DPI (Kumar *et al.*, 2015; Helliwell, 2023)

Table S6 Direct hydrogen bonds and water-mediated hydrogen-bond interactions of GOL of monomer B in the catalytic site of BGl1

**Atom**	**Protein Atom**	**Distance (Å)**[a]	**Angle (º)**
Gol 501B O1	Gol501B O2	2.9 (0.07)	0.0
	Asn162B ND2	2.7 (0.07)	147.5
	Glu361B OE2	2.6 (0.07)	104.6
Gol 501B O2	Gln17B OE1	2.7 (0.07)	125.2
	Gol501B O3	3.0 (0.08)	0.0
	His118B NE2	2.9 (0.07)	162.2
	Trp416B NE1	3.0 (0.07)	162.1
Gol 501B O3	Glu415B OE2	2.8 (0.08)	115.8
	Wat673C O	3.0 (0.09)	0.0
	**Total 9**		

[a] The numbers in parentheses show the uncertainty standard values of the distance. The uncertainty values were calculated taking into consideration the individual uncertainty values (coordinate error) of each atom involved in the interaction, based on DPI (Kumar *et al.*, 2015; Helliwell, 2023)

Table S7 Van der Waals Interactions of GOL of monomer B in the catalytic site of BGl1

Atom	Protein Atom	Distance (Å)[a]
Gol 501B C1	Gol501B C3	2.5 (0.08)
	Glu163B OE1	3.6 (0.07)
	Gol501B O3	2.8 (0.08)
	Wat673C O	3.3 (0.09)
	His118B NE2	3.9 (0.07)
	Gol501B O2	2.4 (0.08)
	Glu163B CD	3.6 (0.07)
	Glu163B OE2	3.4 (0.07)
	Glu361B CD	3.6 (0.07)
	Glu361B OE1	3.0 (0.07)
	Asn162B ND2	3.9 (0.07)
	Glu361B OE2	3.5 (0.07)
Gol501B O1	Gol501B C3	3.8 (0.08)
	Glu163B OE1	3.7 (0.07)
	Gol501B C2	2.4 (0.08)
	His118B NE2	3.2 (0.07)
	Glu163B CD	3.4 (0.07)
	Glu163B OE2	3.3 (0.07)
	Asn301B ND2	3.6 (0.07)
	Glu361B CD	3.2 (0.07)
	Glu361B OE1	3.0 (0.07)
	His118B CE1	3.4 (0.07)
	Asn162B CG	3.4 (0.07)
	Asn162B OD1	3.3 (0.08)
Gol501B C2	Gln 17B OE1	3.8 (0.07)
	Trp408B CZ2	3.7 (0.07)
	Gol501B O3	2.4 (0.08)
	His118B NE2	3.8 (0.07)
	Trp416B NE1	4.0 (0.07)
	Trp408B CE2	3.9 (0.07)
	Trp408B CH2	3.9 (0.07)
	Glu361B CD	3.9 (0.07)
	Glu361B OE1	3.3 (0.07)
	Glu361B OE2	3.7 (0.07)
Gol501B O2	Gol501B C3	2.4 (0.08)

	Gln17B CD	3.5 (0.07)
	Gln 17B NE2	3.8 (0.07)
	Trp408B CZ2	3.8 (0.07)
	His118B CD2	4.0 (0.07)
	Trp416B CE2	3.7 (0.07)
	Trp416B CZ2	3.8 (0.07)
	Trp408B CH2	3.7 (0.07)
	His118B CE1	3.7 (0.07)
Gol501B C3	Trp408B NE1	3.8 (0.07)
	Glu415B OE1	3.5 (0.08)
	Glu415B OE2	3.7 (0.08)
	Trp408B CZ2	3.7 (0.07)
	Trp416B NE1	4.0 (0.07)
	Trp108B CE2	3.7 (0.08)
	Glu361B OE1	3.9 (0.08)
Gol501B O3	Glu415B CD	3.6 (0.08)
	Glu415B OE1	3.5 (0.08)
	Trp416B CD1	3.6 (0.08)
	Trp416B NE1	3.5 (0.08)
	**Total 54**	

[a] The numbers in parentheses show the uncertainty standard values of the distance. The uncertainty values were calculated taking into consideration the individual uncertainty values (coordinate error) of each atom involved in the interaction, based on DPI (Kumar *et al.*, 2015; Helliwell, 2023)

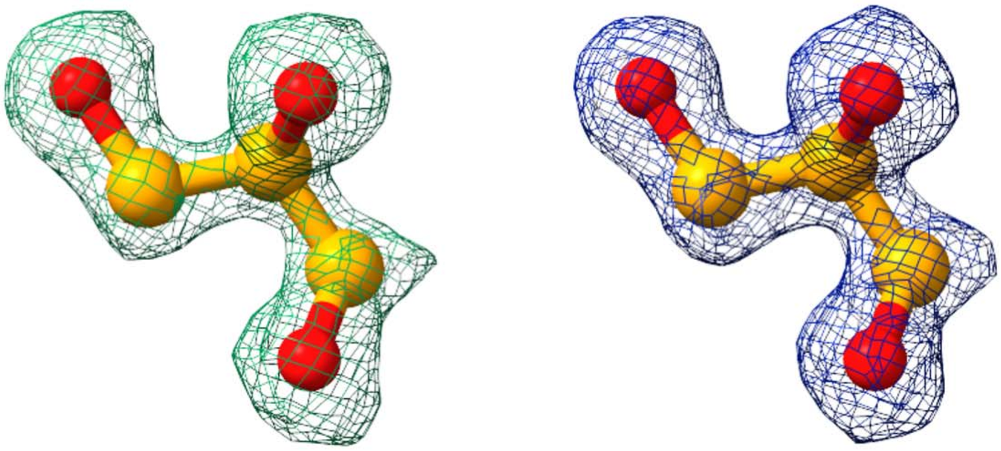

Figure S4 Schematic representation of the unbiased F_{obs}-F_{calc} (green density: left) and 2F_{obs}-F_{calc} (blue density: right) electron density maps contoured at 3.0 σ and 1.0 σ level, respectively, indicating the position of the glycerol molecule bound at the catalytic site of Bgl1.

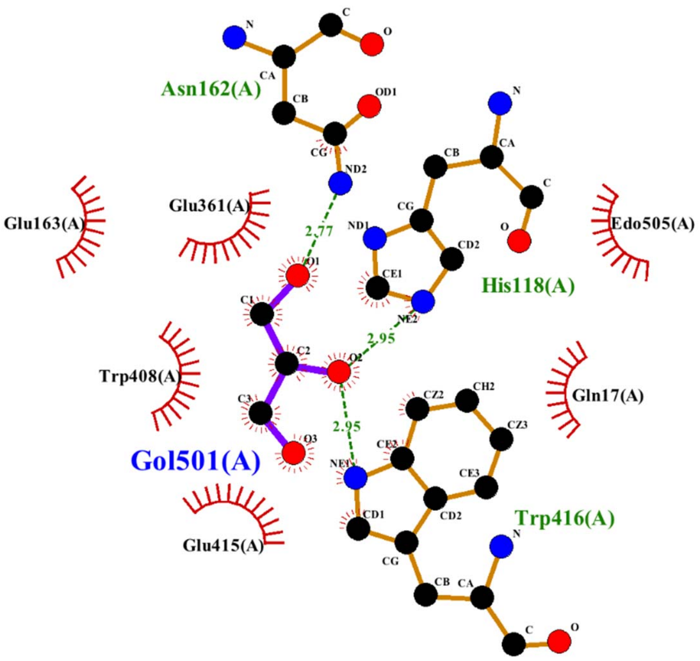

Figure S5 LIGPLOT diagram of GOL interacting with the active-site residue lying in the vicinity. The substrate-analogue bonds are shown in purple, while bonds of the residues lining the site are shown in black. Hydrogen bonds are shown as black dashed lines with distances indicated in A˚.

Table S8 Direct hydrogen bonds and water-mediated hydrogen-bond interactions of EDO in the catalytic site of BGl1

**Atom**	**Protein Atom**	**Distance (Å)**	**Angle (º)**
Edo505A O1	Edo505A O2	3.08	0.0
	Glu163A OE2	2.65	113.2
Edo505A O2	Gol501A O3	2.93	0.0
	Glu163A OE1	2.83	126.1

Table S9 Van der Waals Interactions of EDO in the catalytic site of BGl1

**Atom**	**Protein Atom**	**Distance (Å)**
Edo505A C1	Tyr303A CD2	3.69
	Tyr303A CE3	3.42
	Trp334A CH2	3.89
	Edo505A O1	1.42
	Edo505A C2	1.48
	Edo505A O2	2.41
	Glu163A OE1	3.88
	Glu163A OE2	3.51
Edo505A O1	Tyr303A CD2	3.62
	Tyr303A CE2	3.50
	Edo505A C2	2.43
	Edo505A O2	3.08
	Glu163A CD	3.34
	Glu163A OE1	3.39
	Glu163A OE2	2.65
	Asn219A CB	3.80
Edo505A C2	Gol501A O3	3.59
	Edo505A O2	1.42
	Wat788C O	3.30
	Glu163A OE1	3.59
Edo505A O2	Gol501A O3	2.93
	Gol501A C1	3.29
	Gol501A C3	3.96
	Glu163A CD	3.70
	Glu163A OE1	2.83
	Glu163A OE2	3.79
	Trp119A CH2	3.72
